# Patient outcomes following after-hours and weekend admissions for cardiovascular disease in a tertiary hospital in Calabar, Nigeria

**DOI:** 10.5830/CVJA-2016-025

**Published:** 2016

**Authors:** Victor Ansa, Uchenna Njideoffor, Charles Nworah,, Clement Odigwe, Akaninyene Otu, Affiong Oku

**Affiliations:** Cardiology Unit, Department of Internal Medicine, University of Calabar, Calabar, Cross River State, Nigeria; Cardiology Unit, Department of Internal Medicine, University of Calabar, Calabar, Cross River State, Nigeria; Cardiology Unit, Department of Internal Medicine, University of Calabar, Calabar, Cross River State, Nigeria; Cardiology Unit, Department of Internal Medicine, University of Calabar, Calabar, Cross River State, Nigeria; Department of Internal Medicine, University of Calabar, Calabar, Cross River State, Nigeria; Department of Community Medicine, University of Calabar, Calabar, Cross River State, Nigeria

**Keywords:** cardiovascular disease, After hours, weekend, Calabar, poor outcome, staffing

## Abstract

**Background:**

There are various reports of higher mortality rates occurring after admissions over the weekend and during after-hours. This study aimed to determine if there was a difference in mortality rates occurring during the weekend and after-hours among cardiovascular admissions in a tertiary hospital in Nigeria.

**Methods:**

A review of cardiovascular admissions (including stroke) was carried out at the University of Calabar Teaching Hospital in Nigeria from January 2010 to December 2013. All admissions to the medical wards from the emergency department and medical out-patient department clinics during the study period were included.

**Results:**

A total of 339 patients were studied and stroke was the commonest type of cardiovascular disease (CVD) admitted (187; 55.2%). Hypertension was the commonest cause of heart failure (70; 48.6%). Presentation to hospital during after-hours and length of stay of more than 14 days were significant predictors of death (OR: 3.37; 0.22).

**Conclusion:**

An increase in CVD mortality rates occurred during after-hours, most likely a consequence of uneven staffing patterns and poor access to equipment. Healthcare providers in Nigeria need to consider remedies to this with a view to reducing excess mortality rates.

## Background

Staffing of hospitals generally tends to be lower during weekends and after-hours.^1^ After-hours services entail engaging in or operating after the legal or conventional closing time during weekdays. Considerable strain is put on health workers who cover these shifts due to lower staffing in hospitals during these periods. These health personnel also tend to be juniors who work with less supervision. This potentially poorer quality of medical care2 tends to occur irrespective of the fact that the incidence of many medical diseases is similar from day to day.^3^ Also, very ill patients may present during after-hours, as disease appears to be no respecter of time.

Various researchers have demonstrated strong associations between these staffing variations and higher mortality rates during weekends and after-hours.^4^,^6^ A ‘weekend effect’ has been demonstrated in various studies to occur for a variety of diagnoses, including stroke, myocardial infarction, pulmonary embolism and ruptured aortic aneurysm.^1^,^7^,^9^

Delays in the review of patients and in obtaining senior opinions have been suggested as contributing factors to avoidable mortalities at these times.^10^ These findings pose a strong challenge to the concept of equity, which posits that patients receive equal care regardless of when they present to hospital.

The majority of studies on hospital mortality rates during weekends and after-hours have been carried out in developed countries with considerably stronger healthcare systems, compared to developing nations. Very little research has been done in Nigeria to see if the reported increase in mortality rates in hospitals during the weekend and after-hours applies in our context. This assessment is crucial as identification and quantification of increased weekend mortality rates may promote the redesign of healthcare services in order to improve outcomes. Nwosu and colleagues, in their study of in-patient data in all wards of a tertiary hospital in Nigeria, found a significant difference in hospital mortality rates between weekdays and weekends only in patients admitted to the labour ward.^11^

In this study, we aimed to investigate cardiovascular admissions, including stroke, in the University of Calabar Teaching Hospital (UCTH), to determine if there was a significant difference in mortality rates occurring during the weekend or after-hours compared with regular working hours.

## Methods

This retrospective medical record review was carried out in UCTH from January 2010 to December 2013. The UCTH is the only tertiary health facility in the Cross River state, which is in south-eastern Nigeria. It receives referrals from across the state and its environs.

All admissions to the medical wards from the emergency department and medical out-patients’ department clinics during the study period were included. This comprised adults over 18 years of age. Cardiovascular admissions, including strokes, were then extracted. After-hours was considered to be between 16:00 on one day and 08:00 the next day. Weekends were defined as the period from 16:00 on Friday to 08:00 on Monday. All other times were defined as weekdays.

Socio-demographic data such as age, gender, ethnicity, marital status and occupation were captured. Other data such as day and time of admission, clinical diagnosis, and length of stay in hospital, as well as outcome of the admission were also ascertained.

The case definition for cardiovascular disease was any disorder of the heart and/or blood vessels and included the following: coronary heart disease, cerebrovascular disease, peripheral arterial disease, rheumatic heart disease, congenital heart disease, arrhythmias, deep-vein thrombosis and pulmonary embolism, and their complications. Ethical clearance was obtained from the Health Research and Ethics Committee (HREC) of the UCTH.

## Statistical analysis

Data were analysed using STATA V 13.0 (Stata Corp Lp, College of Station Texas, USA). Continuous variables were presented as means or median and interquartile ranges (for skewed data), while categorical variables were presented as percentages. The chi-squared test was used to test the significance of association between categorical variables. Continuous variables were also converted into categorical variables and compared using the chi-squared test (or Fischer’s exact test where indicated). A logistic regression model was built to identify covariates of poor outcome among the patients studied. A p-value of < 0.05 was regarded as the level of statistical significance.

## Results

A total of 339 patients were admitted during the study period and this accounted for 34.5% of the total medical admissions. Most (286; 84.4%) of these admissions occurred via the emergency department while 53 (15.6%) came via the medical out-patients’ clinic. Females were in the majority (207; 61.1%) with a male:female ratio of 1:1.05 (p = 0.92). The median age of all participants was 55 years with an interquartile range of 47–65 years. The commonest occupational category among the patients was skilled non-manual (120; 35.4%), while professional workers were fewest (7; 2.1%), as shown in Table 1.

**Table 1:** Socio-demographic characteristics of the subjects

*Variable*	*Frequency*	*Percentage*
Age (years)		
< 29	15	4.4
30-39	36	10.6
40-49	47	13.9
50-59	104	30.7
≥ 60	137	40.4
Gender		
Male	132	38.9
Female	207	61.1
Occupation		
Professional	7	2.1
Managerial	53	15.6
Skilled manual	49	14.5
Skilled non-manual	120	35.4
Retired	95	28.0
Student	15	4.4

**Fig 1.**
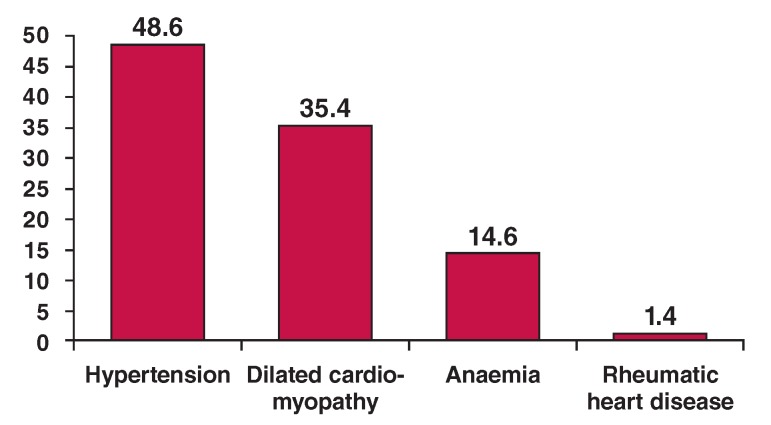
Causes of heart failure.

With regard to the type of cardiovascular disease (CVD) diagnosed on presentation to hospital, stroke was most common (187; 55.2%) and this was closely followed by congestive heart failure (CHF) in 144 (42.5%) patients. The causes of heart failure included hypertension (70; 48.6%), dilated cardiomyopathy (51; 35.4%), rheumatic heart disease (2; 1.4%) and anaemia (21; 14.6%) (Fig. 1).

More patients (257; 75.8%) presented during weekdays A compared with over the weekends, as shown in Table 2. Of the 339 patients studied, slightly more than half (198; 58.4%) presented during working hours, while 141 (41.6%) presented during after-hours. The consultant’s review of the studied patients tended to occur mostly within two to seven days of admission (54%) and few patients (23; 6.8%) were reviewed by consultants more than seven days after admission, as shown in Table 2. In 4% of all patients studied, a consultant was the first contact doctor. More patients (54.4%) were admitted for less than 14 days, compared with those admitted for a longer time.

**Table 2:** Profile of cardiovascular disease/presentation

*Variable*	*Frequency*	*Percentage*
Type of cardiovascular disease		
Stroke	187	55.2
Heart failure (CHF)	144	42.5
Arrhythmias	8	2.4
Causes of heart failure		
Hypertension	70	48.6
Dilated cardiomyopathy	51	35.4
Anaemia	21	14.6
Rheumatic heart disease	2	1.4
Time of presentation		
Weekdays	257	75.8
Weekends	82	24.2
Working hours	198	58.4
After hours	141	41.6
First contact doctor		
Consultant	14	4.0
Senior resident	6	1.8
Registrar/house officer	319	98.2
Days to consultant review		
≤ 1	133	39.2
2-7	183	54.0
> 7	23	6.8
Days admitted		
< 14	197	54.4
>14	152	45.6

CHF: congestive heart failure.

Bivariate analysis revealed that being over 55 years of age, type of CVD, rheumatic heart disease and anaemia as causes of heart failure, as well as duration of hospitalisation were significantly associated with poor outcome (p < 0.05), as shown in Table 3. Further subgroup analysis revealed that patients with CHF had a higher risk of a poor outcome compared with those who had CVD due to stroke or arrhythmias. Variables that were not significantly associated with poor outcome were gender, status of first contact doctor, mode of admission, time of day seen, day of presentation and promptness of consultant’s review.

**Table 3:** Bivariate analysis of variables with regard to poor outcome

*Variable*	*Died (%)*	*Dischargedl referred*	*LAMA (%)*	*Chi-squared test*	*p-value*
Age (years)					
≤ 55	29 (16.1)	129 (71.7)	22 (12.2)	6.17	0.04*
> 55	43 (27.0)	101 (63.5)	15 (9.4)		
Gender					
Male	28 (21.2)	91 (68.9)	13 (9.8)	0.26	0.88
Female	44 (21.3)	139 (67.1)	24 (11.6)		
Type of CVD					
Heart failure (CHF)	18 (12.5)	110 (76.4)	16 (11.5)	15.6	0.004*
Stroke	54 (28.9)	113 (60.4)	20 (10.7)		
Arrhythmias	0 (0)	7 (87.5)	1 (12.5)		
First contact doctor					
Consultant	2 (14.3)	11 (78.6)	1 (1.7)	3.75	0.23
Senior resident	0 (0)	6 (100)	0 (0)		
Registrar/HO	70 (21.9)	213 (66.8)	36 (11.3)		
Admission route					
ED	64 (22.4)	189 (66.1)	33 (11.5)	2.75	0.25
MOPD	8 (15.1)	41 (77.4)	4 (7.5)		
Time seen					
After hours	35 (24.8)	89 (63.1)	17 (12.1)	2.53	0.28
Working hours	37 (18.7)	141 (71.2)	20 (10.1)		
Presented					
Weekday	55 (21.4)	173 (67.3)	29 (11.3)	0.19	0.91
Weekend	17 (20.7)	57 (69.5)	8 (9.8)		
Consultant’s review					
≤ 1 day	35 (26.3)	81 (60.9)	17 (12.8)	4.91	0.09
> 1 day	37 (18.0)	20 (9.7)			
Days admitted					
≤ 14	56 (28.4)	113 (57.4)	28 (14.2)	23.8	< 0.001*
> 14	16 (11.3)	117 (82.4)	9 (6.3)		
Heart failure causes (n = 144)					
Hypertension	9 (12.8)	55 (78.6)	6 (8.6)		
Dilated cardiomyopathy	1 (2.0)	44 (86.2)	6 (11.8)		
Rheumatic heart disease	1 (50.0)	1 (50.0)	0 (0)	18.7	0.004*
Anaemia	7 (33.4)	10 (47.6)	4 (19.0)		

LAMA: left against medical advice, ED: emergency department, MOPD: medical out-patients’ department

A logistic regression model was built using seven variables, as show in Table 4. Presentation to hospital during after-hours and hospital stay of more than 14 days were significant predictors of poor outcome. Those who presented to hospital after hours were three times more likely to have a poor outcome, compared to those who presented within regular working hours. Also, patients who were admitted for more than 14 days had a greater likelihood of having a poor outcome, compared with those who spent less than 14 days in hospital.

**Table 4:** Predictors of poor outcome among all patients

**	**	*95% confidence*	**
*Variable*	*Odds ratio*	*interval*	*p-value*
Age (years)			
≤ 55	1.89	0.73–4.86	0.19
> 55	1		
Gender			
Female	1.05	0.44–2.47	0.92
Male	1		
Route of admission			
ED	1.01	0.31–3.34	0.99
MOPD	1		
Presentation time			
After hours	3.37	0.31–0.56	0.04*
Working hours	1		
Day presented			
Weekday	1.10	0.38–3.21	0.86
Weekend	1		
Causes of heart failure			
Other	0.83	0.33–2.05	0.68
Hypertension	1		
Duration of admission (days)			
≤ 14	0.22	0.08–0.59	0.003*
14>	1		

ED: emergency department, MOPD: medical out-patients’ department.

## Discussion

This study has shown that most patients with CVD presenting at our centre in a developing country were in the sixth decade of life, which is two decades earlier than the typical presentation in developed countries. This corroborates the findings of the INTERHEART Africa study.^12^

Most of the patients were in the lower middle class (skilled non-manual), the so called ‘urban poor’, who have absorbed the Western lifestyle as a status symbol, an indication of the epidemiological transition currently taking place in this region. There were fewer professionals, in the higher socio-economic class, and this could have been attributed to their better awareness of cardiovascular risk factors and the adoption of healthy lifestyles, or better compliance with their medication. Another reason may have been that professionals do not commonly use public hospitals such as the one in which this study was carried out.

The majority of the patients presented with stroke and this corroborates the findings in other studies, which have shown that stroke is more common in black hypertensives than in non-blacks,^13^,^14^ and that the risk of a first stroke is about twice as high in blacks as in whites.^15^ Stroke has been identified as a major health problem in Nigeria,^16^,^18^ which could be linked to a high incidence of severe hypertension owing to poor compliance with medication and lifestyle-modification strategies. Often in our setting, however, the patient may even be unaware of the presence of hypertersion.^19^ The exact mechanism for the higher frequency of stroke in blacks remains unclear.

In our series, most patients presented during weekdays and the majority during working hours. This pattern of presentation may be attributed to the preference of patients or caregivers to present at these times with the hope that they will receive better care. Weekends and after hours are often characterised by the constraints of understaffing and poor access to specialised services.^10^

This study revealed that the odds of dying were significantly higher among those who presented during after-hours, compared with those who presented during working hours. Therefore for every 10 deaths among patients admitted during after-hours, we would expect three among patients admitted during normal working hours. Being hospitalised for more than 14 days was also a predictor of mortality. However, being admitted over the weekend was not found to be a predictor of mortality.

The significantly higher mortality rate registered during afterhours may have been multifactorial. There are usually fewer workers during after-hours and these tend to be juniors with less clinical experience. Some of these may be filling in for regular staff and may not have a good knowledge of the patients and the internal workings of the particular unit. Handing-over sessions may not be effectively implemented, resulting in serious gaps in clinical knowledge that may adversely affect crucial management decisions. Also, there tends to be fewer supervisors during after-hours to provide oversight in various clinical scenarios.^20^,^21^ Ancillary services such as laboratories and radiology, which provide crucial support in the management of critically ill patients, are usually less accessible during after-hours. These factors may all have contributed to higher mortality rates being recorded during after-hours.

Interestingly, the higher mortality rate was not recorded when weekend admissions were compared with weekday admissions. It has been noticed in our hospital that admission rates tend to decline over the weekends. This may be due to the perception within the populace that only skeletal services can be obtained over the weekends. It is possible that the more critically ill patients are taken to private hospitals during the weekend instead of being brought to our centre. This might account for the lack of difference between in-hospital mortality rates during weekends compared with weekdays.

The higher mortality rates among those who were in hospital for more than 14 days may have been linked to disease severity. It is likely that those who remained in hospital for longer periods suffered from more severe forms of disease that led inexorably to poorer outcomes. This finding is in agreement with a report that highlighted a strong correlation between the high Acute Physiology and Chronic Health Evaluation (APACHE) III and multiple-organ dysfunction syndrome scores and prolonged length of stay for critically ill patients in the intensive care unit.^22^,^23^

Our findings suggest that healthcare providers in Nigeria should consider the potential increase in mortality rate that may arise as a consequence of uneven staffing patterns, especially during after-hours. The economic implications of striving to achieve and maintain a consistent level of staffing naturally come to the fore. Although it has been suggested that maintaining high levels of staffing may sometimes be economical, it is often not feasible. However, innovation is required to ensure that such re-organisation represents an efficient use of scarce resources.

A limitation of this study is that owing to the high cost and sometimes unavailability of facilities for neuro-imaging, the majority of patients with stroke did not have imaging records, so the different types of stroke could not be clearly determined.

## Conclusion

Our findings confirm that outcome is poor for cardiovascular admissions during after-hours but not during weekends. It is suggested that patients may deliberately be avoiding seeking medical care in public institutions during weekends. The increase in CVD mortality may be as a consequence of uneven staffing patterns, especially during after-hours. Healthcare providers in Nigeria should strive to achieve and maintain a consistent level of staffing, especially during after-hours and weekends, despite the economic implications. This is often not feasible, therefore innovation is required to ensure that such re-organisation represents an efficient use of scarce resources.
